# Assessing indirect biodiversity conservation benefits of fisheries closures in the Gulf of St. Lawrence, Canada

**DOI:** 10.1371/journal.pone.0316754

**Published:** 2025-01-09

**Authors:** Andrea Bryndum-Buchholz, Tyler D. Eddy, Jonathan A. D. Fisher

**Affiliations:** Centre for Fisheries Ecosystems Research, Fisheries and Marine Institute of Memorial University, St John’s, Newfoundland and Labrador, Canada; King Fahd University of Petroleum & Minerals, SAUDI ARABIA

## Abstract

Marine biodiversity loss is a pressing global issue, intensified by human activities and climate change. Complementary to marine protected areas (MPAs), Other Effective Area-Based Conservation Measures (OECMs) have emerged as a key tool to mitigate this loss by providing long-term biodiversity protection. However, while OECMs primarily target specific taxa, they can also offer indirect biodiversity conservation benefits (BCBs) to a wider range of taxa. In this study, we assess the indirect BCBs of eleven OECMs in the Gulf of St. Lawrence, focusing on their role in supporting the life-history processes of commercially important species such as Atlantic halibut, Greenland halibut, and redfish. We apply an integrated assessment that combines knowledge and data previously unconnected to provide information to support OECM management. Our analysis reveals that eight of the eleven OECMs overlap with potential spawning habitats for these species, suggesting important life-history benefits. However, projected climate-driven changes in bottom temperature, oxygen concentration, and pH levels pose a threat to these habitats, potentially undermining the long-term effectiveness of OECMs. These findings underscore the need for adaptive management strategies that incorporate climate-informed ecosystem indicators and broaden the conservation focus beyond economically important species. Such approaches are crucial to ensuring that OECMs continue to provide both direct and indirect BCBs in the face of accelerating climate change, thereby contributing to global marine biodiversity conservation efforts.

## Introduction

Marine biodiversity loss is a critical global concern, particularly in a world of rapid environmental changes driven by human activities and climate change [1, 2]. To address this issue, international agreements have pledged to protect 30% of the ocean by 2030 (the 30x30 agenda) [3]. Among the tools, developed to address this challenge are Marine Protected Areas (MPAs), Marine Reserves, and Other Effective Area-Based Conservation Measures (OECMs) [4]. While MPAs are specifically designed to protect biodiversity, OECMs are not designed to do, but are rather intended to provide long-term, in-situ protection for biodiversity as one outcome, also referred to as biodiversity conservation benefits (BCBs) [5]. For example, OECMs in Canada are established under the *Fisheries Act* and are essentially fisheries closures [6], which often do not have management plans specifically intended for biodiversity conservation benefits, but rather focus on fisheries [6, 7]. While the primary objective of OECMs is direct BCBs to specific taxa, these areas can also offer important indirect BCBs to a wider range of taxa [4, 8, 9]. In Canada, many OECMs have been established to safeguard vulnerable benthic species such as cold-water corals and sponges [9]. While these closures provide direct BCBs to these species, they may also offer indirect BCBs for other species that rely on that habitat for completion of their life cycles, including spawning and development of early life stages [4, 10].

One such area is the Gulf of St. Lawrence (henceforth, also referred to as the Gulf), a highly productive region in Atlantic Canada. The Gulf is a semi-enclosed sea whose seafloor is a mosaic of deep channels, shallow banks, sediment-covered areas, and unique ecosystems like cold-water coral reefs and sponge fields [11, 12]. Notably, the Laurentian Channel which runs through the Gulf, reaches depths of over 500 meters in some places [12]. The Magdalen Shallows are a notable submarine bank, which are relatively shallow compared to the surrounding waters [12]. Such banks are important fishing grounds due to the aggregation of marine species and serve as feeding areas for many organisms [13]. The Gulf is home to cold-water coral reefs and sponge-dominated benthic habitats, particularly along the deeper parts of the Laurentian Channel. These ecosystems are sensitive and considered biodiversity hotspots [10, 14].

The Gulf’s high concentration of cold-water corals and sponges that create complex habitats that support a wide range of species, including those that rely on these structures for feeding, protection, and reproduction [9]. For example, the important fisheries species Atlantic halibut (*Hippoglossus hippoglossus*), Greenland halibut (*Reinhardtius hippoglossoides*), and redfish (*Sebastes* spp.) depend on these specific deep-water habitats for spawning and early life stages [15–17]. The overlap between OECMs in the Gulf of St. Lawrence, established to protect corals sponges and sea-pens, with potential spawning habitats of these three fish species presents an opportunity to assess the indirect BCBs provided by OECMs, specifically under current and future environmental conditions.

The provision of BCBs by OECMs is essential for sustaining long-term marine biodiversity [8, 9]. However, recent studies have documented rising bottom temperatures and decreasing oxygen concentration in the region, trends that are expected to continue and intensify throughout the 21^st^ century [18, 19]. These changes are likely to exceed the tolerance thresholds of the three focus species, leading to shifts in their distribution and potentially reducing the effectiveness of OECMs in providing indirect BCBs [20, 21]. Moreover, ocean acidification is expected to further degrade the structural complexity of cold-water coral and sponge habitats, potentially diminishing their capacity to support marine biodiversity in the region [22]. The risk of ineffective marine biodiversity conservation through OEMCs in Canada and beyond has been discussed, both in the context of climate change and the 30x30 agenda (e.g., [6, 7, 23–26].

Research on the indirect BCBs of OECMs, particularly those protecting cold-water coral and sponge habitats in Canada, remains limited [9, 27–29]. This study contributes to filling this gap by documenting the current indirect BCBs provided by a nine, selected OECMs in the Gulf of St. Lawrence (Table 1) and examines their potential to provide these benefits under future climate scenarios. Here, we aim to evaluate the indirect BCBs of selected OECMs established in the Gulf of St. Lawrence in 2017 by adopting an integrated assessment approach. We integrate and analyse data and knowledge from multiple sources not available at the time of OECM establishment to create a comprehensive assessment of OECM outcomes in terms of indirect BCBs and potential climate change impacts on those. The implications of our findings extend beyond the ecological sphere, highlighting consequences OECM policy and management. As the environment continues to change, adaptive management will be crucial to ensuring that OECMs remain effective in fulfilling their conservation objectives [23]. Through this study, we aim to contribute to a deeper understanding of the role of OECMs in marine biodiversity conservation and to provide insights that can inform future conservation efforts in the face of a changing climate.

**Table 1:** Overview of Other Effective Conservation Measures (OECMs) in the Gulf of St. Lawrence included in this study with direct and indirect biodiversity conservation benefits (BCBs).

ID	OECM	Appr. Size [km^2^]	Av. Depth ± St.Dev [m]	Direct BCB	Indirect BCBs	Reference
1	Beaugé Bank Sponge Conservation Area	215	86.63 ± 9.86	Cold-water corals	Protection of habitat used by fish and invertebrates.	[30]
2	Central Gulf of St Lawrence Coral Conservation Area	1,284	405.81 ± 14.64	Sea pens		[31]
3	East of Anticosti Island Sponge Conservation Area	939	132.35 ± 26.35	Cold-water corals		[32]
4	Eastern Gulf of St Lawrence Coral Conservation Area	423	462.02 ± 17.45	Sea pens		[33]
5	Eastern Honguedo Strait Coral and Sponge Conservation Area	2,338	366.12 ± 22.46	Cold-water corals & Sea pens		[34]
6	Jacques-Cartier Strait Sponge Conservation Area	346	97.87 ± 18.56	Cold-water corals		[35]
7	North of Bennett Bank Coral Conservation Area	821	401.54 ± 25.35	Sea pens		[36]
8	Parent Bank Sponge Conservation Area	530	122.91 ± 40.86	Cold-water corals		[37]
9	Slope of Magdalen Shallows Coral Conservation Area	335	388.79 ± 19.23	Sea pens		[38]
10	South-East of Anticosti Island Sponge Conservation Area	845	357.96 ± 34.28	Cold-water corals		[39]
11	Western Honguedo Strait Coral Conservation Area	496	377.93 ± 6.64	Sea pens		[40]

## Material and methods

### Knowledge and data layers

We synthesized data sources on eight selected OECMs, associated species, and variables for the bottom environment in the Gulf (details on the specific data sets and their respective sources are in Tables 1 and 2): (i) marine conservation management—spatially explicit boundaries and properties of current significant benthic habitats and OECMs; (ii) species tracking and geolocation modeling for Atlantic halibut (*Hippoglossus hippoglossus*); (iii) systematic field sampling and DNA identification of Greenland halibut (*Reinhardtius hippoglossoides*); (iv) systematic field sampling of redfish larvae (*Sebastes spp*); (v) life-history parameters in terms of preferred temperature ranges for non-spawning and spawning adults of Atlantic halibut, Greenland halibut, and redfish; (vi) High-resolution, future climate change projections of bottom temperature (°C), bottom oxygen concentration (mmol m^-3^), and bottom pH, under two climate change emissions scenarios, Shared Socioeconomic Pathways (SSPs)—SSP2-45 and SSP5-85. We selected these particular variables due to their associations with the geographic distribution and life-history traits of demersal species, including spawning locations and successful completion of early life stages (e.g., [15, 17, 41–43]). The two pathways, SSP2-45 and SSP5-85, translate to moderate to extreme changes in the three bottom environment variables: increase in bottom temperature, decrease in bottom oxygen concentration, decrease in bottom pH [44].

**Table 2:** Data sets, data types, methods and variables combined to analyze indirect current and future biodiversity conservation benefits of Other Effective Conservation Measures (OECMs) in the Gulf of St. Lawrence. SSP = Shared Socioeconomic Pathways; CMIP6: Couple Inter-model Intercomparison Project 6.

Data set	Data types, methods and variables		Source
OECMs	Shapefiles of OECM boundaries with the following attributes: Name of area, area size (km^2^), primary biodiversity conservation objective, prohibitions within area, location, and weblink to each area profile. From these weblinks, we derived information on the indirect biodiversity conservation objectives.		[45]
Significant benthic habitats	Shapefiles of significant benthic habitats for cold-water sponges and sea pens, with information of location and size of habitat (km^2^). These habitats were identified using Kernel density estimation and species distribution modeling. This method creates a modelled biomass surface for each taxon and an aerial expansion method permitted to identify significant concentrations of sponges and sea pens. The borders of the areas were refined using knowledge of null catches and species distribution models. Predictive models were produced using a random forest machine-learning technique.		[46–48]
Potential Atlantic halibut spawning habitat	Likelihood (probability from 0–1) of Atlantic halibut spawning habitat in the Gulf of St. Lawrence, based on reconstructed movement tracks, derived from pop-up satellite archival tags, using a statistical geolocation Hidden Markov model. Likelihood is represented as the number of individual spawning events per grid cell (defined as location of tagged individual) during 2014–2016 and 2018 (winters).		[49]
Potential Greenland halibut spawning habitat	Potential spawning area for Greenland halibut in the Gulf of St. Lawrence, based information from ichthyoplankton and bottom trawl surveys during 2005–2009.		[50, 51]
Potential redfish spawning habitat	Density and distribution of redfish larva in the Gulf of St. Lawrence based on plankton sampling between 22 June and 4 July 1991 and at 74 stations, and between 10 June and 20 June 1992.		[52]
Preferred temperature ranges for non-spawning and spawning adults	Redfish	Non-spawning: 3–8 °C	[53, 54]
		Spawning: 3.7–6.2 °C	
	Greenland halibut	Non-spawning: -1.9–9 °C	[55, 56]
		Spawning: 2.8–4.1 °C	
	Atlantic halibut	Non-spawning: -1.5–15 °C	[15, 57]
		Spawning: 5–7 °C	
Climate change projections	Decadal, present day (2010–2020) and future projections of (2050–2060; 2090–2100) mean conditions along the sea bottom for temperature (°C), oxygen concentration (mmol m^3^), and bottom pH. Future conditions projected under SSP2-45 and SSP5-85. Data for present day conditions are based on Global Ocean Physics Reanalysis and Forecast and the Global Ocean Biogeochemistry Analysis and Forecast provided by the Copernicus Marine Environment Monitoring Service (https://data.marine.copernicus.eu/products); future conditions are based on an Earth System Model ensemble (incl. ACCESS-ESM1-5; CanESM5; CESM2-WACCM; CNRM-ESM2-1; GFDL-ESM4; GISS-E2- 1-G; IPSL-CM6A-LR; MIROC-ES2L; MPI-ESM1- 2-LR; UKESM1- 0-LL) from CMIP6, downscaled to a spatial resolution of 0.05°.		[58]

### Data analyses

#### Spatio-temporal changes in the bottom environment

We extracted spatially resolved (0.05° x 0.05° grid cell resolution) ensemble mean projections for bottom temperature, oxygen concentration, and pH from the Bio-ORACLE ERDDAP server (https://tinyurl.com/Bio-ORACLEv3). All variables of the Bio-ORACLE v.3.0 dataset were assessed for their reliability and accuracy through cross-validation against in-situ observation from the Global Ocean Data Analysis Project [58]. Further, we selected mean decadal projections for present-day conditions (2010–2019) as well as future conditions (2050–2059 and 2090–2099) under two emissions scenarios (SSP2-45 and SSP5-85) from the Bio-ORACLE v.3.0. dataset. Next, to analyse future changes in environmental variables across the Gulf of St. Lawrence and for each OECM, we standardized decadal changes to relative change in 2050–2059 (mid-century) and 2090–2099 (end-century) relative to the 2010–2019 (baseline). Lastly, based on percent relative change of the bottom environmental variables, we calculated the mean percent change in each variable by OECM, emissions scenario, and future timeframe. All analyses were conducted in R Studio (version 2023.12.0+369) with R 4.4.0.

#### Spatial overlap analysis

We combined the data layers (Table 2) and the relative changes in the bottom variables to assess overall environmental changes in the Gulf and to evaluate whether potential indirect BCBs from the selected OECMs occur currently. By identifying which areas may experience the least/most environmental changes by mid-, and end-century, we also assessed whether current, indirect BCBs are likely to persist with intensifying climate change or not.

## Results

### Current OECMs and spawning habitats in the Gulf of St. Lawrence

**Fig 1:**
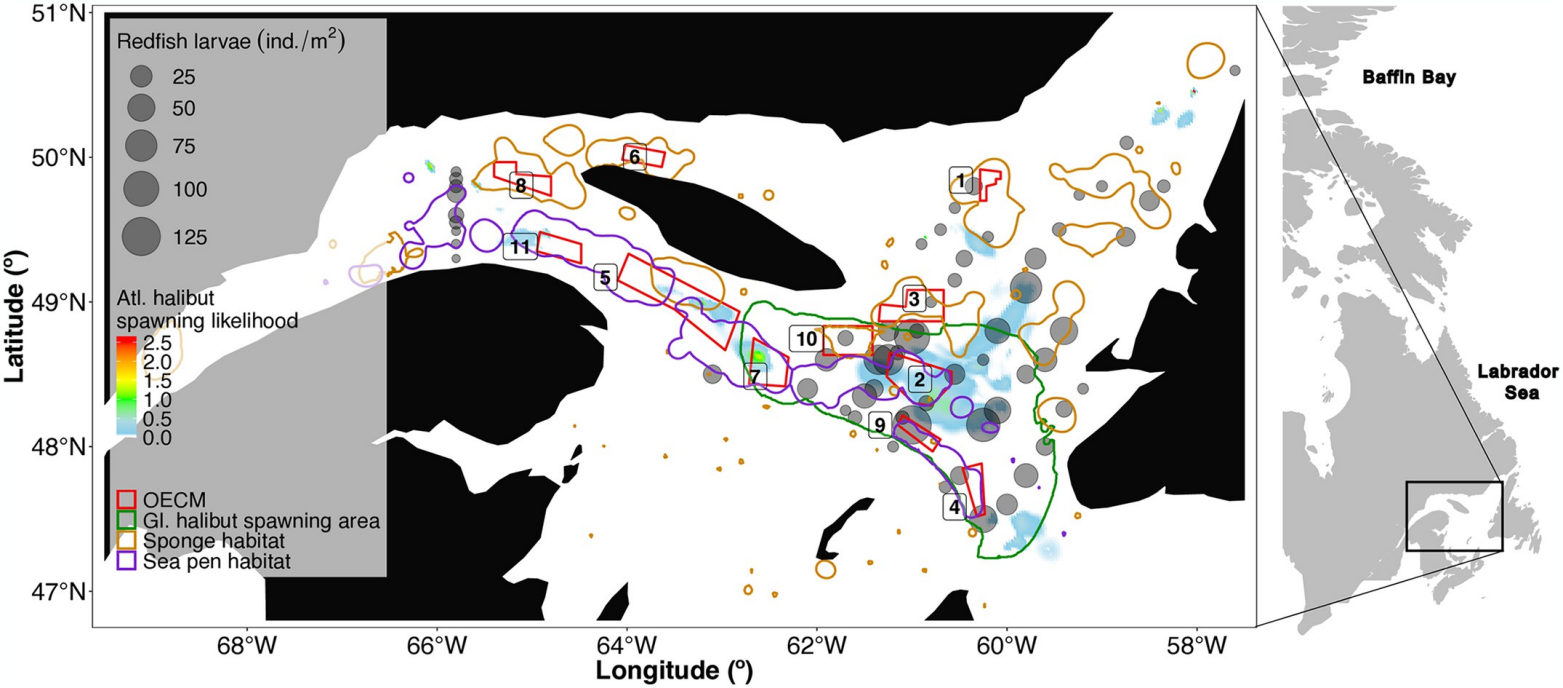
Overlap of current Other Effective area-based Conservation Measures (OECMs) in the Gulf of St. Lawrence with significant benthic habitats and potential spawning habitat for three demersal fish species. Significant benthic habitat includes habitat for cold-water sponges and sea pens [46, 47]; potential spawning areas include areas for Greenland halibut (*Reinhardtius hippoglossoides*; 50,51), Atlantic halibut *(Hippoglossus hippoglossus*; [49]), and redfish (*Sebastes mentella*, *Sebastes fasciatus*; [52]). Land shapefiles retrieved from http://www.naturalearthdata.com/; shapefiles for OECM outlines retrieved from [45].

Based on the current potential spawning habitat of Atlantic halibut, Greenland halibut, and redfish (henceforth, also referred to as focus species) were largely located in the Laurentian Channel and overlapped with current OECMs (Fig 1). 5 out of 11 OECMs (Central Gulf of St Lawrence Coral Conservation Area, Eastern Gulf of St Lawrence Coral Conservation Area, North of Bennett Bank Coral Conservation Area, Slope of Magdalen Shallows Coral Conservation Area, and South-East of Anticosti Island Sponge Conservation Area) overlapped with proposed spawning habitat of Greenland halibut (Fig 1). The Central Gulf of St Lawrence Coral Conservation Area had the highest likelihood of harbouring Atlantic halibut spawning habitat with a range of 0.06–0.12 (on a scale of 0–1; Table 2), followed by the North of Bennett Bank Coral Conservation Area, the Slope of Magdalen Shallows Coral Conservation Area, and the Western Honguedo Strait Coral Conservation Area (Fig 1 and S1 Fig). The highest redfish larvae density (100–120 ind./m^2^) overlapped with the Central Gulf of St Lawrence Coral Conservation Area, the Eastern Gulf of St Lawrence Coral Conservation Area, and the Slope of Magdalen Shallows Coral Conservation Area. The potential spawning habitats for all fish species overlapped with vulnerable benthic habitats for sponges and sea pens (Fig 1).

### Current and future bottom environment of the Gulf of St. Lawrence

**Fig 2:**
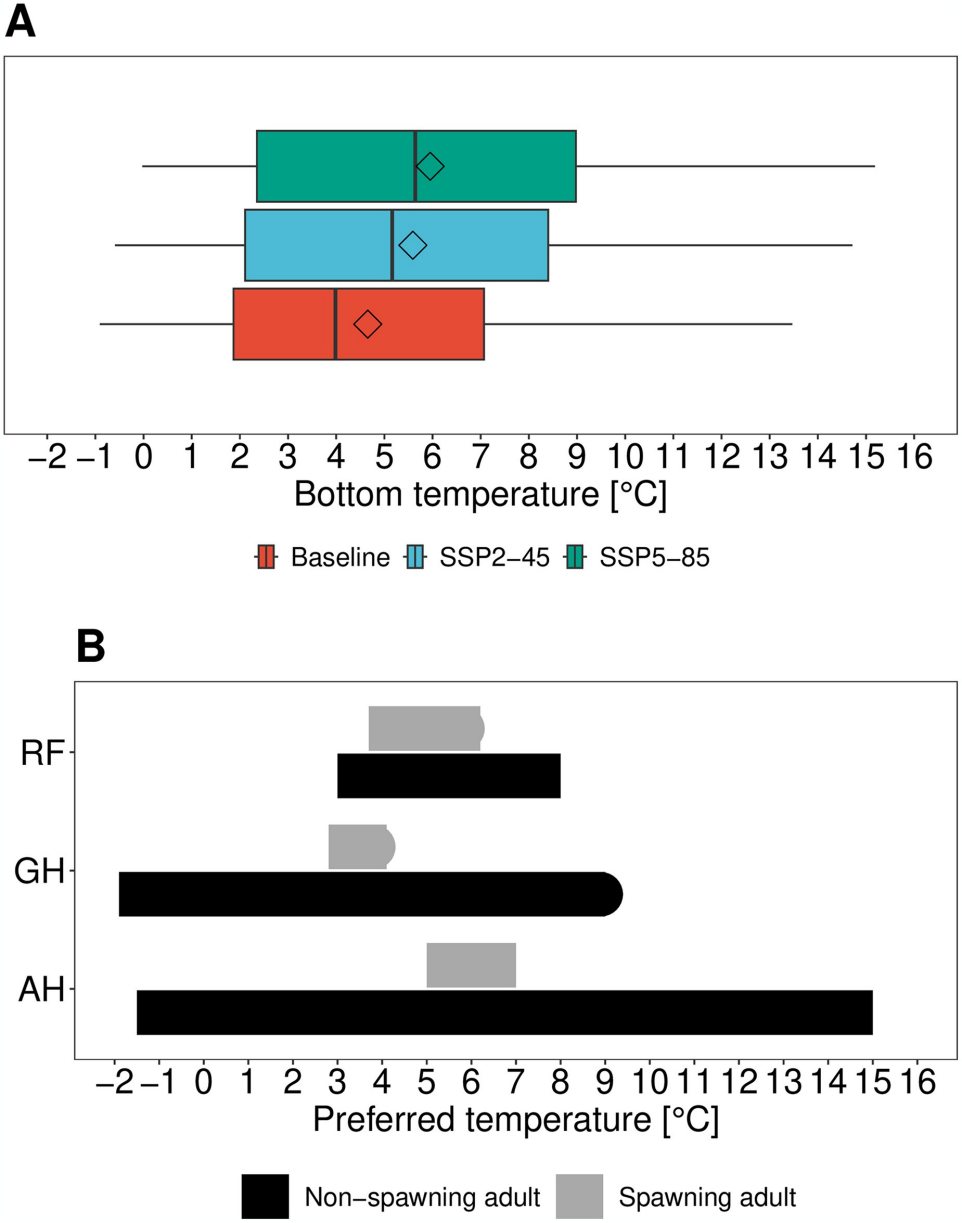
Bottom temperatures in the Gulf of St. Lawrence and preferred temperature ranges for three demersal fish species. **(A)** Baseline (2010–2019) and projected (2050–59, under SSP2-45, SSP5-85) bottom temperature variability, mean, and median across the Gulf of. St. Lawrence. Boxplots: upper and lower hinges correspond to the first and third quartiles; upper/lower whiskers extend to the highest/lowest value within 1.5 times the interquartile range; horizontal lines within boxes correspond to the median; diamonds represent the mean; outlier dots represent data beyond the end of the whiskers. **(B)** Preferred temperature ranges for spawning and non-spawning adults of redfish (RH; *Sebastes mentella*, *Sebastes fasciatus*), Greenland halibut (GH; *Reinhardtius hippoglossoides*), and Atlantic halibut (AH; *Hippoglossus hippoglossus*). Analysis for changes for 2090–99 are in S2 Fig.

Mean bottom temperature across the Gulf of St. Lawrence from 2010–2019 (henceforth, referred to as baseline) was 4.33 °C; under the mid-century future projections (2050–2059), mean bottom temperatures were between 5.57 °C (SSP2-45) and 5.95 °C (SSP5-85) (Fig 2). The mean baseline and the mid-century temperatures under both SSPs lay within the preferred temperature ranges for non-spawning adult Atlantic halibut (-1.5–15 °C), redfish (3–8 °C), and Greenland halibut (-1.9–9 °C). For spawning adults, the preferred temperature range is narrower compared to non-spawning adults. Mean baseline and future bottom temperature across the Gulf of St. Lawrence was within the preferred temperature of adult spawning redfish and Atlantic halibut; adult Greenland halibut is likely to face unfavourable spawning habitat conditions in the future (Fig 2). While mean temperature largely remained within preferred temperature ranges of the three focus species, the high variability of the projected temperature under both SSPs across the Gulf also indicates that these thresholds may be seasonally exceeded (Fig 2 and S2 Fig). This pattern was exacerbated with intensifying future climate change, as represented by the end-century changes under SSP5-85, where the projected mean bottom temperature is exceeding the preferred temperature for spawning adults across all three focus species (S2 Fig).

**Fig 3:**
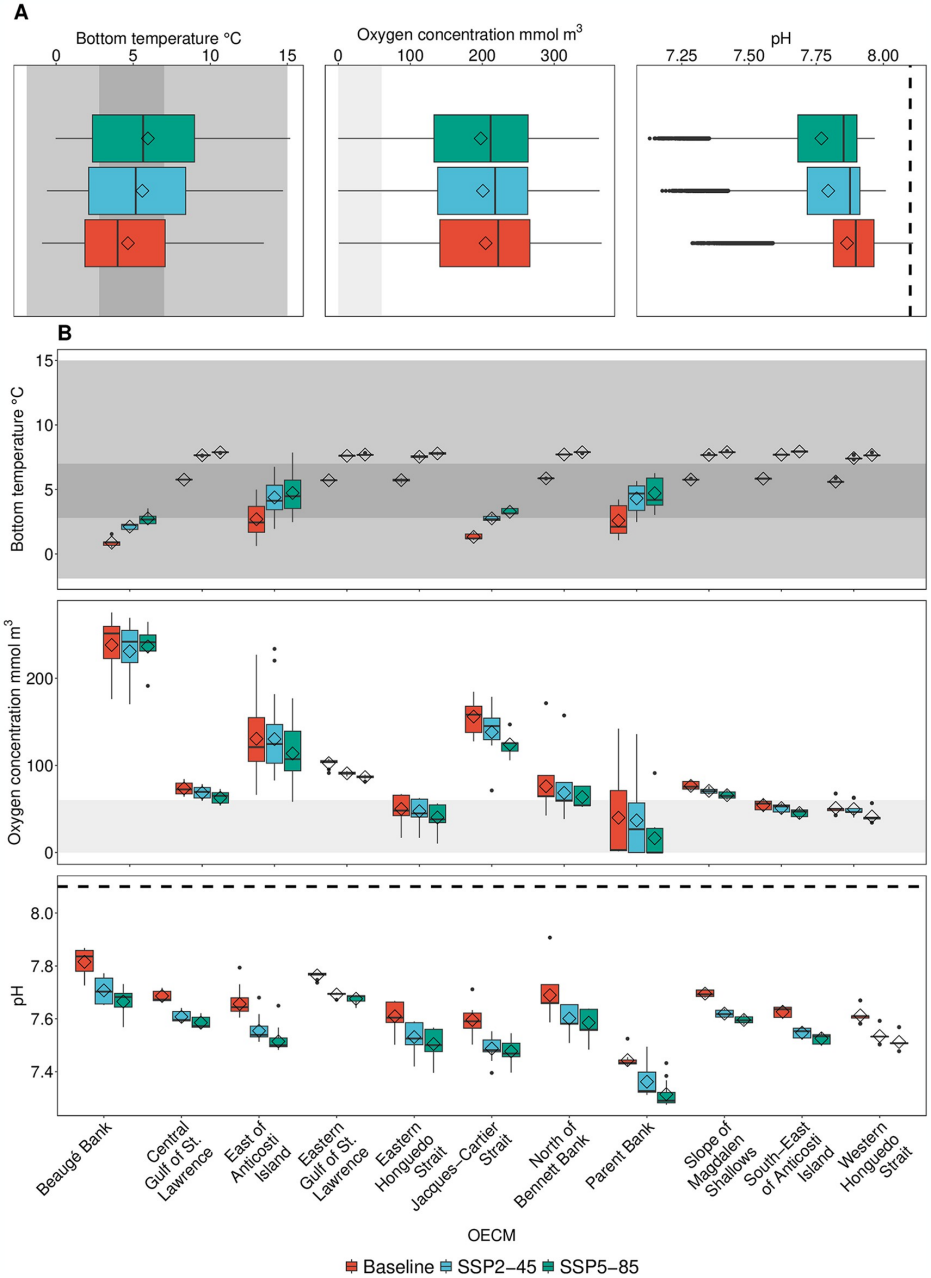
Baseline and mid-century, future bottom sea temperature (°C), bottom oxygen concentration (mmol m^3^), and pH; (A) across the Gulf of St. Lawrence and (B) within individual Other Effective area-based Conservation Measures (OECMs). Baseline values include the years 2010–2019; future projections include the time frame 2050–2059 under two shared socioeconomic pathways (SSPs). Dark grey shading indicates the preferred temperature range for adult non-spawning (grey) and spawning (darkgrey) Atlantic halibut (*Hippoglossus hippoglossus*), Greenland halibut (*Reinhardtius hippoglossoides*), and redfish (*Sebastes mentella*, *Sebastes fasciatus*); values are from FishBase (https://www.fishbase.se/search.php). Light grey shading indicates oxygen concentration below 60 mmol m^3^, the threshold for a marine ecosystem to be considered hypoxic [20]. Dashed black line indicates today’s average ocean pH [59]. Boxplots: upper and lower hinges correspond to the first and third quartiles; upper/lower whiskers extend to the highest/lowest value within 1.5 times the interquartile range; horizontal lines within boxes correspond to the median; diamonds represent the mean; outlier dots represent data beyond the end of the whiskers. Baseline and end-century changes are shown in S3 Fig.

The impact of changing bottom temperature on non-spawning and spawning adults, and by extension on the provision of long-term BCBs, may be exacerbated by changes in decreasing bottom oxygen concentrations and increasing pH (Fig 3). This becomes especially apparent when zooming into the individual OECMs (Fig 3B). For all OECMs, mean baseline bottom temperature was largely within the preferred temperature range for spawning adults of the three focus species (Fig 3B). Mid-century, projected temperature changes under both emissions scenarios remained only within this range for the East of Anticosti Island, Jacques-Cartier Strait, and Parent Bank; all other OEMCs were projected to experience temperature increase exceeding the species-specific preferred spawning temperature (Fig 3B). This pattern is exacerbated by the end of the 21^st^ century (S3 Fig).

Mean bottom oxygen concentration across the OECMs was largely above the hypoxia threshold for the baseline and mid-century time periods (Fig 3B). Mid-century mean oxygen concentration was below the hypoxia threshold within the Eastern Honguedo Strait, Parent Bank, South-East of Anticosti Island, and Western Honguedo Strait, and the South-East of Anticosti Island Sponge Conservation Area (Fig 3B). By the end of the 21^st^ century, these areas remain within hypoxic conditions, with the Central Gulf of St. Lawrence and North of Bennet Bank moving into hypoxia as well (S3 Fig).

All OECMs showed mean pH values smaller than the global mean of 8.1, with mid-century future values consistently lower than the baseline values (Fig 3B). Notably, the Parent Bank Sponge Conservation Area showed the lowest pH values across all time frames. This pattern is exacerbated by the end of the 21^st^ century (S3 Fig). Notably, the Parent Bank Sponge Conservation Area showed a pH value a magnitude lower than the global mean under SSP5-85 (pH = 7.1).

### Overlap of OECMs and spawning habitats under future bottom environment changes

**Fig 4:**
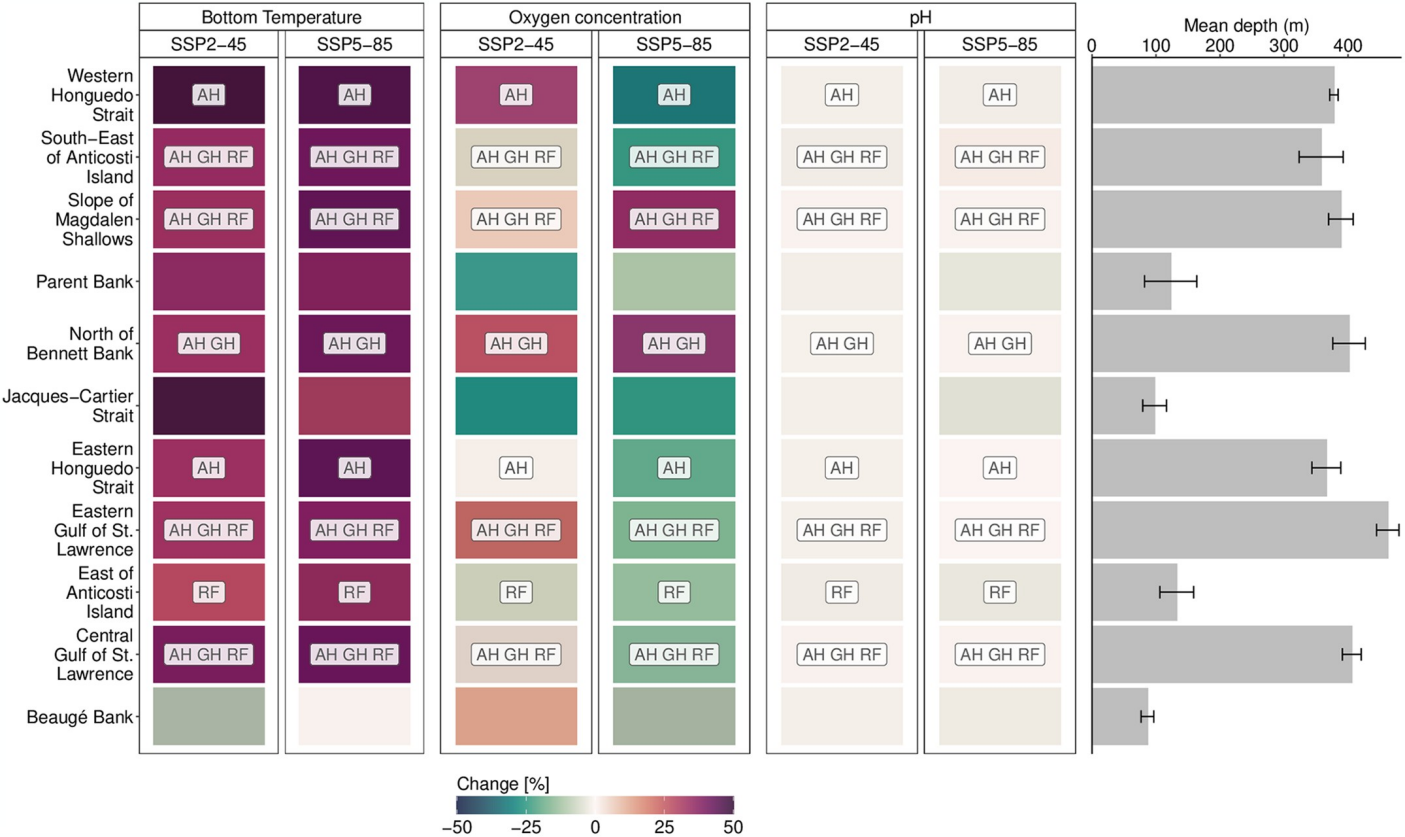
Mid-century percent change in bottom sea temperature, bottom oxygen concentration and pH for individual Other Effective area-based Conservation Measures (OECMs) and associated average depth (m) of each OECM. Changes represent values in 2050–2059 relative to the baseline time frame 2010–2019. Labels indicate whether an OECM is overlapping with potential spawning habitat of AH = Atlantic halibut, GH = Greenland halibut, and RF = redfish. Error bars indicate one standard deviation of the mean. Values for end-century changes are in S4 Fig.

By mid-century, eight out of the eleven OECMS overlapped with potential spawning habitat of at least one of the focus species (Fig 4). Four OECMs overlapped with all three focus species: The Central and Eastern Gulf of St. Lawrence Conservation Areas, the Slope of Magdalen Shallows and the South- East of Anticosti Island Conservation Areas. Interestingly, the Central and Eastern Gulf of St. Lawrence Conservation Areas are the two deepest areas. OECMs that were not overlapping with any potential spawning habitat are the shallowest areas, which is in line with the reproductive behaviour of all three focus species. However, these areas, and potentially the three focus species, were impacted by climate-driven environmental changes with the magnitude of changes varying by depth, SSP and projection time frame (Fig 4 and S4–S6 Figs).

Notably, mid-century bottom temperature under both SSPs increased between 25–50% across all OECMs, except for Beaugé Bank (Fig 4). Under SSP5-85, the South-East of Anticosti Island as well as the Eastern and Central Gulf of St. Lawrence stood out, as they overlapped with all three focus species and are projected to experience simultaneous changes in bottom temperature (≥ 50% increase), bottom oxygen concentration (≤ -25% decrease) and bottom pH (≤ 1% decrease). Interestingly, Beaugé Bank showed the smallest changes in all bottom variables in mean oxygen concentration under both SSPs; however, this area did not overlap with any of the proposed spawning sites of the three focus species. Mean pH levels decreased by mid-century across all OECMs, with Beaugé Bank showing the largest decrease, followed by the Central Gulf of St. Lawrence, which overlaps with potential spawning habitat of all three focus species, and the East of Anticosti Island, which overlapped with potential redfish spawning habitat (Fig 4). By the end of the 21^st^ century, projected changes in the three bottom variables were exacerbated in their direction of change across all OECMs, challenging the long-term provisioning of BCBs under intensifying climate change (S5 and S6 Figs).

## Discussion

We assessed indirect biodiversity conservation benefits of selected OECMs in the Gulf of St. Lawrence using an integrated assessment approach. Our analysis showed that eight out of the eleven OECMs currently overlap with potential Atlantic halibut, Greenland halibut, or redfish spawning habitat, indicating a life-history benefit for these species. OECMs that are not overlapping with any potential spawning habitat are the shallowest areas, which are, however, also important for early life stages of Atlantic halibut [60]. All OECMs are projected to be impacted by future, climate-driven changes in bottom temperature, bottom oxygen concentration, and bottom pH, that may threaten life history dynamics and early-life stage development of the three focal species and highlight climate vulnerability of the region, its species, and the continuing provision of indirect BCBs.

### Current and future provisioning of biodiversity conservation benefits (BCBs)

The main purpose of OECMs is to provide long-term BCBs. While BCBs are the cornerstone of OECM objectives globally, research on indirect BCBs of coral and sponge OECMs across Canada remains limited [9]. Assessing BCBs of OECMs through data and knowledge synergy brings needed insights in terms of these OECM outcomes. Through integrating different assessments of potential Atlantic halibut, Greenland halibut, and redfish spawning sites in the Gulf of St. Lawrence, we documented current indirect BCBs of the existing OECMs in the region. The existence of these indirect BCBs is further supported by the underlying habitat. All OECMs in the Gulf of St. Lawrence are protecting benthic habitats under DFO’s *Sensitive Benthic Areas Policy* and include sensitive cold-water coral and sponge aggregations. Cold-water corals and sponges create habitat for numerous marine species, particularly in regions without natural geographic features that offer structure for fish and invertebrates, such as the Gulf and the Laurentian Channel more specifically [10]. The structures formed by corals and sponges provide various benefits, i.e., enhanced feeding opportunities, protection from predators, spawning and nursery areas, spawning aggregation sites, and attachment points for fish eggs [9, 10].

Whether the current BCBs will prevail under future climate change conditions is uncertain. The OECMs that overlap with the potential spawning cites presently have preferred temperature ranges for all three adult and non-spawning fish species and are above the hypoxia threshold [20]. However, the preferred temperature ranges for adult spawners are at least an order of magnitude smaller than for non-spawners (Fig 2B), rendering their spawning behaviour vulnerable to climate warming. Already, record-breaking bottom temperatures in the Laurentian Channel have been documented [18], with a Gulf-wide average of 6.7 °C, and 7.7°C in the Cabot Strait. Alongside, average oxygen concentration in the Gulf of St. Lawrence has shown a decreasing trend over the last decade, especially in the deeper waters of the Gulf (i.e., the Laurentian Channel), having decreased from ~100–150 μM/kg in 1990s to ∼ 75–100 μM/kg in the early 2020s [19]. As the measurements from the 2020s are largely from samples between 200–250 m, average oxygen concentration may be even lower in depths beyond 250 m, as shown in our baseline model data of deeper OECMs i.e., Central and Eastern Gulf of St. Lawrence Conservation Areas, North of Bennet Bank, and Slope of Magdalen Shallows.

Further, all OECMs under all time frames are showing signs of ocean acidification, indicating future implications for indirect BCBs, not only through impacts on habitat forming species but also through synergistic effects of changing bottom temperature and oxygen concentration on the life-history of our selected demersal fish species [43]. While current research suggests that cold-water corals, such as *Lophelia pertusa* [22, 61] and sponges, such as *Porifera spp*. [62, 63], can still grow under ocean acidification, the overall habitat can undergo significant structural loss providing less complexity and support for biodiversity [22]. This could also impact the continuing provision of spawning habitat and nursery habitats the OECMs in the Gulf of St. Lawrence for redfish [52, 64], Atlantic halibut [49], and Greenland halibut [50, 51]. As bottom temperature increases simultaneously with decreasing oxygen concentration and pH, species will face an increasingly insufficient oxygen supply. This is due to higher metabolism demands with rising water temperature, eventually negatively impacting growth and reproduction, among others [43, 65]. Acidification acts as another stressor on a specie’s metabolism, overall, leading to an reduced resilience to environment changes and long-term survival [43, 66].

Consequently, our results highlight potential losses of indirect BCBs in terms of changing habitat conditions that could affect continued spawning aggregations, larval survival, and growth [17, 67, 68]. However, indirect BCBs could also be challenged by shifting distributions of the three focus species in response to climate-driven environmental changes. We observed the large climate-impacts on the deeper waters of the Laurentian Channel, which begets the question where the redfish, Atlantic halibut and Greenland halibut of the Gulf will find refuge, aggregate for successful spawning and larval development with intensifying climate change—deeper and colder waters as climate-refuges are not available in the Gulf in the future. However, Atlantic halibut and Greenland halibut have already been observed to shift towards northern latitudes in response to warming waters in the Northwest Atlantic [15, 41, 67]; redfish abundance in the Gulf of St. Lawrence, on the other hand, has been increasing significantly in the past decade [17]. However, their habitat within the Gulf of St. Lawrence is projected to become unavailable by the end of the 21^st^ century, due to unfavourable changes in, among others, bottom temperature and bottom oxygen concentration [15, 69]. Whether redfish in the Gulf will also shift their distribution north remains to be seen.

### Implications for fisheries in the Gulf of St. Lawrence

Our results point toward wider implications for associated fisheries that rely on successful spawning and recruitment, as well as historically consistent geographical distribution of the three groundfish species [67, 70]. A prominent example of largely climate-induced changes to a fishery in the region is the recent increase in redfish which has prompted the fishery to be reopened after a ~30-year moratorium [17]. However, given our results, the projections mentioned above, and a recent DFO study [17], the redfish fishery is unlikely to persist over the long-term, as biomass is likely to decrease, independent of fishing pressure. This is partly due to a reduction in the size-at-maturity and recruitment, possibly as a response to the warming bottom waters in the Gulf (redfish grow slower in warmer waters) [9]. However, there is uncertainty in these patterns, as they also showed persistent survival even at 10 °C, indicating that redfish may reside here under future climate change conditions.

While recent rising water temperatures have also been linked to increasing abundance of Atlantic halibut [15, 16] suggest a shift in the Atlantic halibut spawning timing due to these temperature changes, potentially leading to an earlier spawning season in the Gulf of St. Lawrence. This, in turn, could have future consequences for larval survival and recruitment, partly due to a mismatch between food requirements and food availability, affecting subsequent recruitment to the Atlantic halibut fishery [70, 71]. If this is the case, indirect BCBs for Atlantic halibut in the respective OECMs will likely become weaker or disappear with ongoing climate change.

### Implications for OECM policy, designation, and monitoring

Our analysis of indirect BCBs in the present day and throughout the 21^st^ century can inform OECM policy, including OECM designation and monitoring. OECMs are designed to operate similarly to marine protected areas, but they are not officially or legislatively designated as such. Although biodiversity conservation might not be the primary management goal of an OECM, it must be a primary outcome [8, 9]. We show that the majority of the OECMs in the Laurentian Channel are fulfilling this outcome by providing indirect BCBs; all the described benefits are, however, unlikely to persist under climate-induced changes in bottom temperature, oxygen concertation and pH. Consequently, OECM policy and management needs to adapt to those changes to remain effective and to continue their contribution to Canada’s commitment to protect marine biodiversity in the long-term.

Further, all the OECMs assessed in our study exist under the *Sensitive Benthic Areas Policy* [72] which prohibits any form of bottom contact fishing (i.e., bottom trawls, dredges, bottom seining, traps, gillnets, and bottom longlines); however, other fishing practices and industries such as oil and gas are allowed, ultimately questioning actual protection of biodiversity in the respective OECMs [7]. These concerns are in the context of the fact that systematic assessments of OECMs as effective contributors to biodiversity conservation are still relatively sporadic. By integrating existing knowledge into a more complete picture of current and future BCBs in Canada’s OECMs, our results contribute to fill this knowledge gap. However, our analysis is based on opportunistic, if irregular sampling of adults and/or larvae of Atlantic halibut, Greenland halibut and redfish to assess their spawning timing and locations. To assess continuing BCBs for these species, and ideally for other species that may indirectly benefit from the OEMCs, non-invasive annual ecological monitoring needs to take place and ecosystem indicators need to be developed.

When designing new OECMs, an ecosystem-based approach, rather than a single species approach, should ideally being pursued. This is necessary to guarantee that in-situ biodiversity conservation, the main objective of OECMs, is being achieved. To do so, ecosystem indicators, which are ideally climate-informed, reflecting both the primary and indirect BCBs need to be developed and directly integrated across OECMs design, monitoring and management [11]. This way, a more comprehensive understanding of the effectiveness, functionality and relevance, as well as climate change vulnerability to help adapt management can be achieved [73]. Examples of biogeochemical ecosystem indicators include temperature, oxygen concentration, pH, and primary productivity. Ecological ecosystem indicators should include the biomass of species related to primary and indirect BCBs [23, 74], such as cold-water coral cover, sponge cover or redfish biomass. Our study represents one approach in integrating available data and knowledge about the ecosystem protected by OECMs in the Gulf of St. Lawrence, to assess BCBs now and with ongoing climate change. This approach could be translated into a standardized ecosystem indicators for the region, by, for example, providing integrated impact maps on a regular schedule, such as shown in Fig 4.

Finally, targeted and adaptive improvement of OECMs in Canada and other regions of the world will rely on understanding of climate vulnerability by species and by region [75]. Consequences, climate vulnerability assessment need to become a key component in OECM designation and evaluation under current and future climate change. Our approach represents one among several approaches [75], where we specifically aimed at filling the gap of matching management and assessment needs in terms of temporal and spatial scale, hence, moving towards operationalizing climate vulnerability assessments for OECMs.

### Limitations and outlook

Our assessment has its limitations in terms of data availability, species focus, and uncertainty in the applied climate change projections of the environmental variables.

*Data limitations*—the data of potential spawning habitat of Atlantic halibut, Greenland halibut and redfish are based on various sampling and tagging studies that only cover 2–5 years that are not overlapping. While this is the currently available data on these specific spawning behaviours in the region, longer time frames would give a better estimate of current spawning habitats and their variability in the Gulf. This underscored the above-mentioned necessity of continuous ecosystem monitoring of OECMs, where, for example, depth-integrated ichthyoplankton sampling could provide data on larval distribution and density within a given region. While this has been challenging for species that reproduce in the winter due to seasonal ice-cover in the Gulf, climate warming has already and is expected to continue to reduce ice-cover in the Gulf in the future [76], providing a wider sampling window.

Our analysis is based on knowledge from peer-reviewed literature and government publications. While these sources provide evidence of e.g., current and future changes in the bottom environment of the Gulf of St. Lawrence or larval density of specific fish species, local knowledge and Indigenous knowledge can provide additional evidence of ecosystem status and changes within OECMs in Canada [25, 77]. This is further relevant, as the Aichi Biodiversity Targets of the Convention on Biological Diversity’s Strategic Plan for Biodiversity acknowledge Indigenous stewardship for areas outside of protected areas, such as OECMs [78]. Here, the Indigenous Circle of Experts in Canada stipulated these areas as Indigenous Protected and Conserved Areas that are Indigenous led with a long-term commitment to biodiversity conservation [77].

*Species focus*—We focused on three groundfish species in our assessment of indirect BCBs of the OECMs in the Laurentian Channel. This focus was due to the recent findings of potential spawning habitats in of the three groundfish species in the Laurentian Channel [49, 51, 52], as well as their economic contribution to the fishing industry in the region [79]. We assumed a positive impact on their spawning habitat in the Gulf of St. Lawrence due to the OECMs; however, both positive and negative unintended consequences of area-based protection are common [80–82]; warranting a wider assessment focus. For example, to other, less economically valuable species that also play a role in a resilient ecosystem functioning and structure, and consequently also for long-term biodiversity conservation (i.e., winter skate (*Leucoraja ocellata*) and capelin (*Mallotus villosus*) [13, 83].

*Uncertainty in the projections*—the climate change projections for bottom temperature, bottom oxygen concentration, and bottom pH were obtained from Bio-ORACLE v. 3.0 [58]. [58] assessed the reliability and accuracy of the Bio-ORACLE v. 3.0 dataset through cross-validation against data from the Global Ocean Data Analysis Project (GLODAP). GLODAP provides in-situ observations for two of our focus variables (Data for pH cross-validation was not available); however, their spatial coverage does not extent across the entire Gulf of St. Lawrence, potentially influencing the accuracy in the region [58]. For the areas in the Gulf covered by GLODAP observations, the accuracy of the average difference between the downscaled ensemble projections and in situ observations were relatively low for temperature (0.141 °C) compared to oxygen concentration (5.222 mmol m^3^) [58]. Hence, oxygen concentration was generally overestimated for the Gulf, potentially leading to a somewhat smaller future impact on BCBs in our analysis.

## Conclusion

Our study emphasizes the critical role that recently established OECMs in the Gulf of St. Lawrence currently play in providing indirect biodiversity conservation benefits (BCBs) for key commercially exploited species Atlantic halibut, Greenland halibut, and redfish. While current OECMs are effectively protecting crucial benthic habitats and seem to be contributing to the life-history success of these species, their long-term effectiveness is increasingly uncertain due to projected climate-driven changes in bottom temperature, oxygen concentration, and pH levels. These environmental shifts threaten the continued provision of indirect BCBs, potentially leading to habitat degradation, which could undermine the overall conservation goals of these areas.

Our findings highlight the need for adaptive OECM management and policy to ensure that these areas continue to fulfill their biodiversity conservation objectives in the long-term. This includes the integration of climate-informed ecosystem indicators and regular non-invasive ecosystem monitoring to assess continuing provision of direct and indirect BCBs. Additionally, broadening the focus beyond economically important species to include a wider array of biodiversity is central for sustaining ecosystem functioning and long-term conservation outcomes. Addressing these challenges is essential for the OECMs in the Gulf of St. Lawrence and beyond this region to remain effective contributors to marine biodiversity conservation in the face of accelerating climate change.

## Supporting information

S1 Fig Spawning likelihood of Atlantic halibut (*Hippoglossus hippoglossus)* in the Gulf of St. Lawrence. Range of Atlantic halibut spawning likelihood within the existing OECMs in the Gulf of St. Lawrence, as calculated by [49]. Numbers represent the OECM ID in Table 1.
(TIF)

S2 Fig Bottom temperatures in the Gulf of St. Lawrence and preferred temperature ranges for three demersal fish species. **(A)** Baseline (2010–2019) and projected (2090–99, under SSP2-45, SSP5-85) bottom temperature range across the Gulf of. St. Lawrence. Boxplots: upper and lower hinges correspond to the first and third quartiles; upper/lower whiskers extend to the highest/lowest value within 1.5 times the interquartile range; horizontal lines within boxes correspond to the median; diamonds represent the mean; outlier dots represent data beyond the end of the whiskers. **(B)** Preferred temperature ranges for spawning and non-spawning adults of redfish (RH; *Sebastes mentella*, *Sebastes fasciatus*), Greenland halibut (GH; *Reinhardtius hippoglossoides*), and Atlantic halibut (AH; *Hippoglossus hippoglossus*). Analysis for changes for 2050–59 are in Fig 2.
(PDF)

S3 Fig Baseline and end-century, future bottom sea temperature (°C), bottom oxygen concentration (mmol m^3^), and pH; (A) across the Gulf of St. Lawrence and (B) within individual Other Effective area-based Conservation Measures (OECMs). Baseline values include the years 2010–2019; future projections include the time frame 2090–2099 under two shared socioeconomic pathways (SSPs). Dark grey shading indicates the preferred temperature range for adult non-spawning (grey) and spawning (darkgrey) Atlantic halibut (*Hippoglossus hippoglossus*), Greenland halibut (*Reinhardtius hippoglossoides*), and redfish (*Sebastes mentella*). Light grey shading indicates oxygen concentration below 60 mmol m^3^, the threshold for a marine ecosystem to be considered hypoxic [20]. Dashed black line indicates today’s average ocean pH [59]. Boxplots: upper and lower hinges correspond to the first and third quartiles; upper/lower whiskers extend to the highest/lowest value within 1.5 times the interquartile range; horizontal lines within boxes correspond to the median; diamonds represent the mean; outlier dots represent data beyond the end of the whiskers.
(TIF)

S4 Fig End-century percent change in bottom sea temperature, bottom oxygen concentration, and pH for individual Other Effective area-based Conservation Measures (OECMs) and associated average depth (m) of each OECM. Changes represent values in 2090–2099 relative to the baseline time frame 2010–2019. Labels indicate whether an OECM is overlapping with potential spawning habitat of AH = Atlantic halibut, GH = Greenland halibut, and RF = redfish. Error bars indicate one standard deviation of the mean.
(TIF)

S5 Fig Projected percent changes for sea bottom temperature, bottom oxygen concentration, and pH by mid-, and end-century (2050–2059 and 2090–99) under SSP2-45 in the Gulf of St. Lawrence. All changes are relative to the baseline condition in 2010–2019. Red outlines denote established Other Effective Conservation Measures (OECMs), retrieved from [45]. Data from Bio-ORACLE v.3.0 [58]. Land shapefiles retrieved from http://www.naturalearthdata.com/.
(TIF)

S6 Fig Projected percent changes for sea bottom temperature, bottom oxygen concentration, and pH by mid-, and end-century (2050–2059 and 2090–99) under SSP5-85 in the Gulf of St. Lawrence. All changes are relative to the baseline condition in 2010–2019. Red outlines denote established Other Effective Conservation Measures (OECMs), retrieved from [45]. Data from Bio-ORACLE v.3.0 [58]. Land shapefiles retrieved from http://www.naturalearthdata.com/.
(TIF)
