# Supplementary figures and images for: Assessing indirect biodiversity conservation benefits of fisheries closures in the Gulf of St. Lawrence, Canada
Source: PLoS One. 2025 Jan 9;20(1):e0316754. doi: 10.1371/journal.pone.0316754 (PMC11717253; doi:10.1371/journal.pone.0316754)

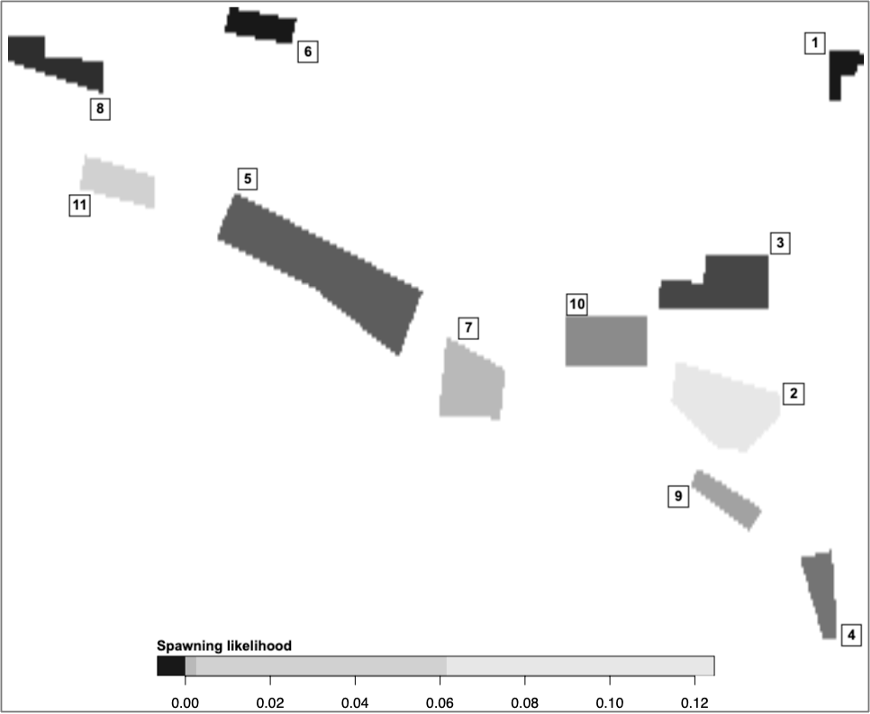

Supplement: S1 Fig — Range of Atlantic halibut spawning likelihood within the existing OECMs in the Gulf of St. Lawrence, as calculated by [49]. Numbers represent the OECM ID in Table 1. (TIF) [file pone.0316754.s001.tif]

**A**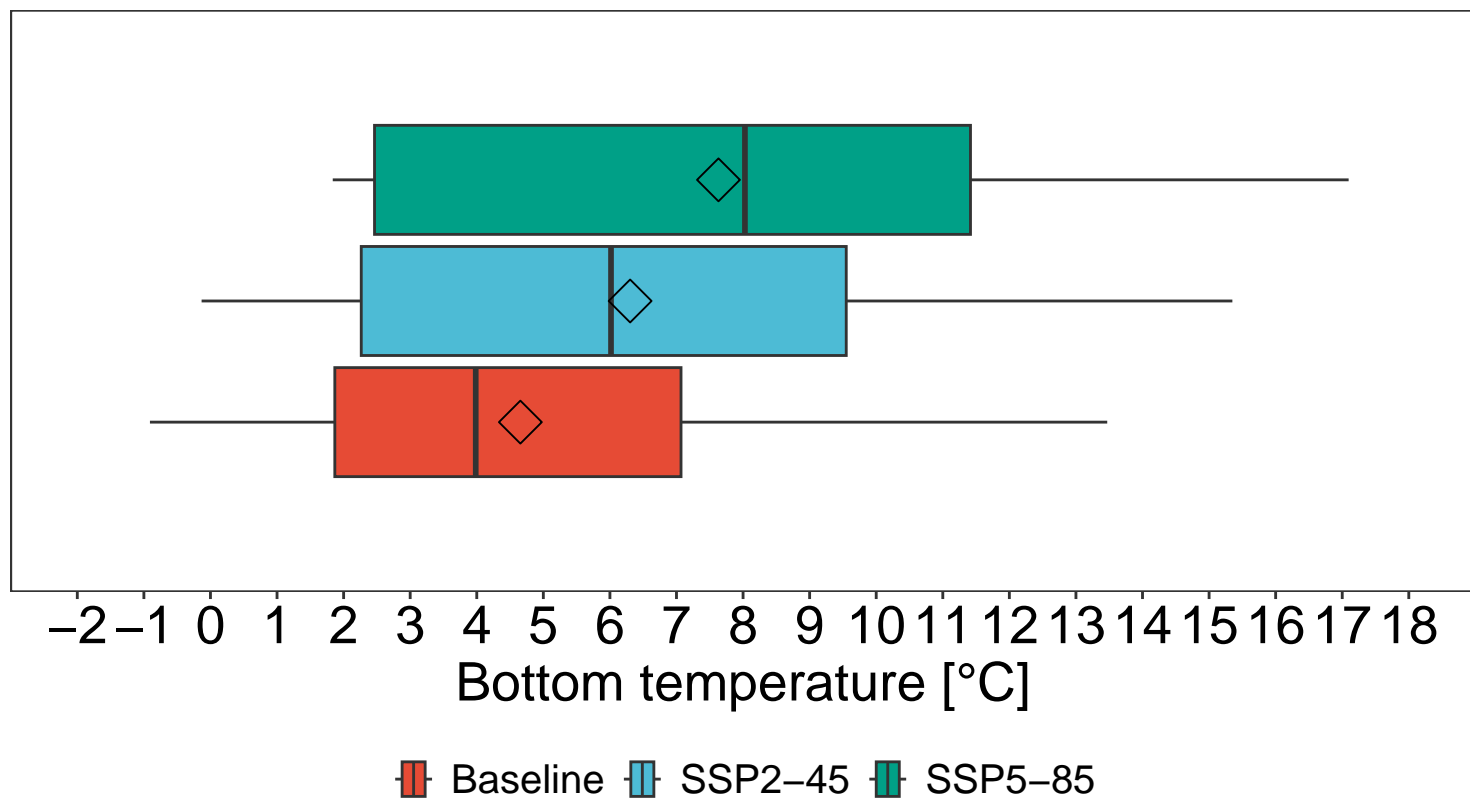**B**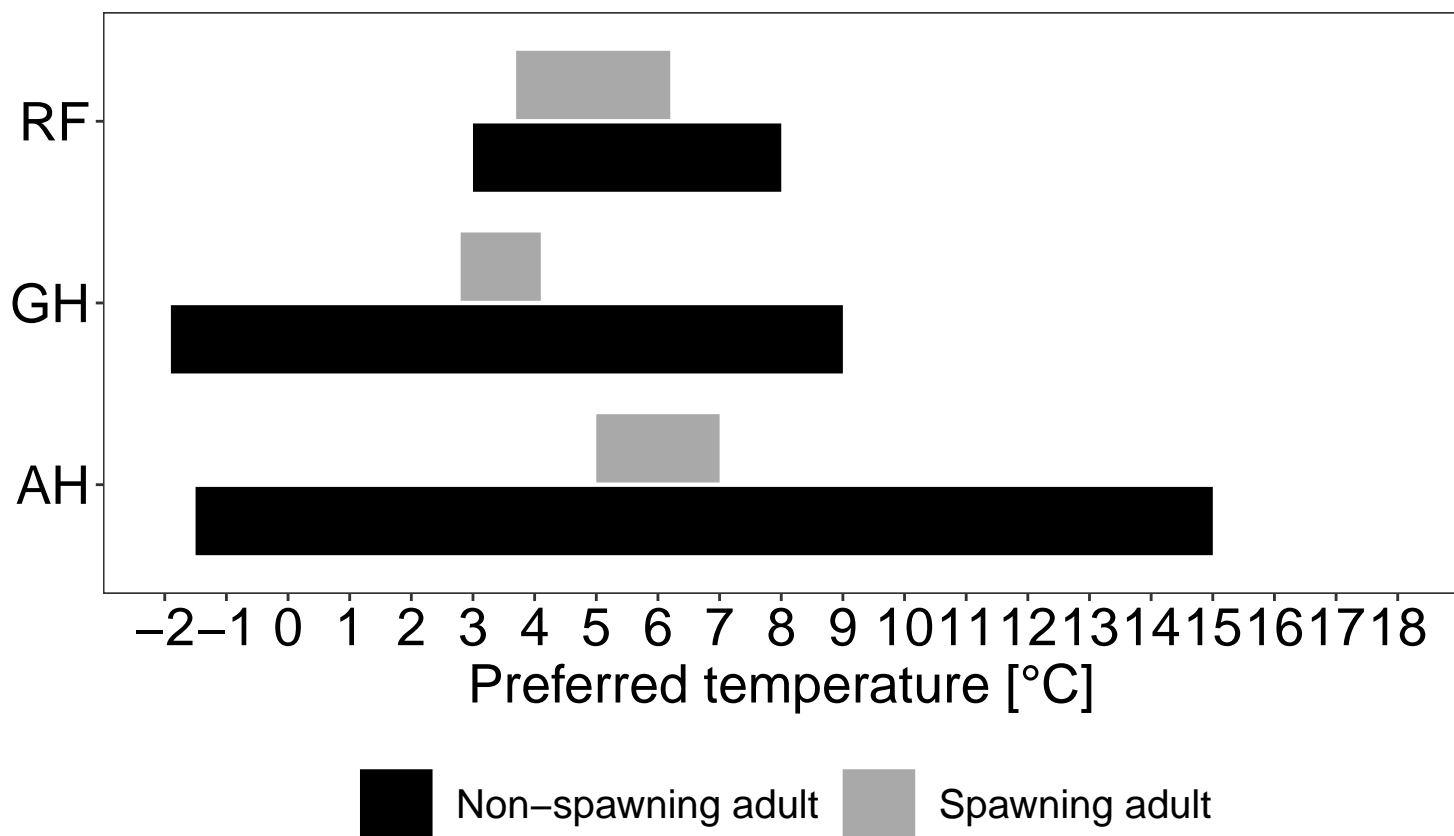

Supplement: S2 Fig — (A) Baseline (2010–2019) and projected (2090–99, under SSP2-45, SSP5-85) bottom temperature range across the Gulf of. St. Lawrence. Boxplots: upper and lower hinges correspond to the first and third quartiles; upper/lower whiskers extend to the highest/lowest value within 1.5 times the interquartile range; horizontal lines within boxes correspond to the median; diamonds represent the mean; outlier dots represent data beyond the end of the whiskers. (B) Preferred temperature ranges for spawning and non-spawning adults of redfish (RH; Sebastes mentella, Sebastes fasciatus), Greenland halibut (GH; Reinhardtius hippoglossoides), and Atlantic halibut (AH; Hippoglossus hippoglossus). Analysis for changes for 2050–59 are in Fig 2. (PDF) [file pone.0316754.s002.pdf]

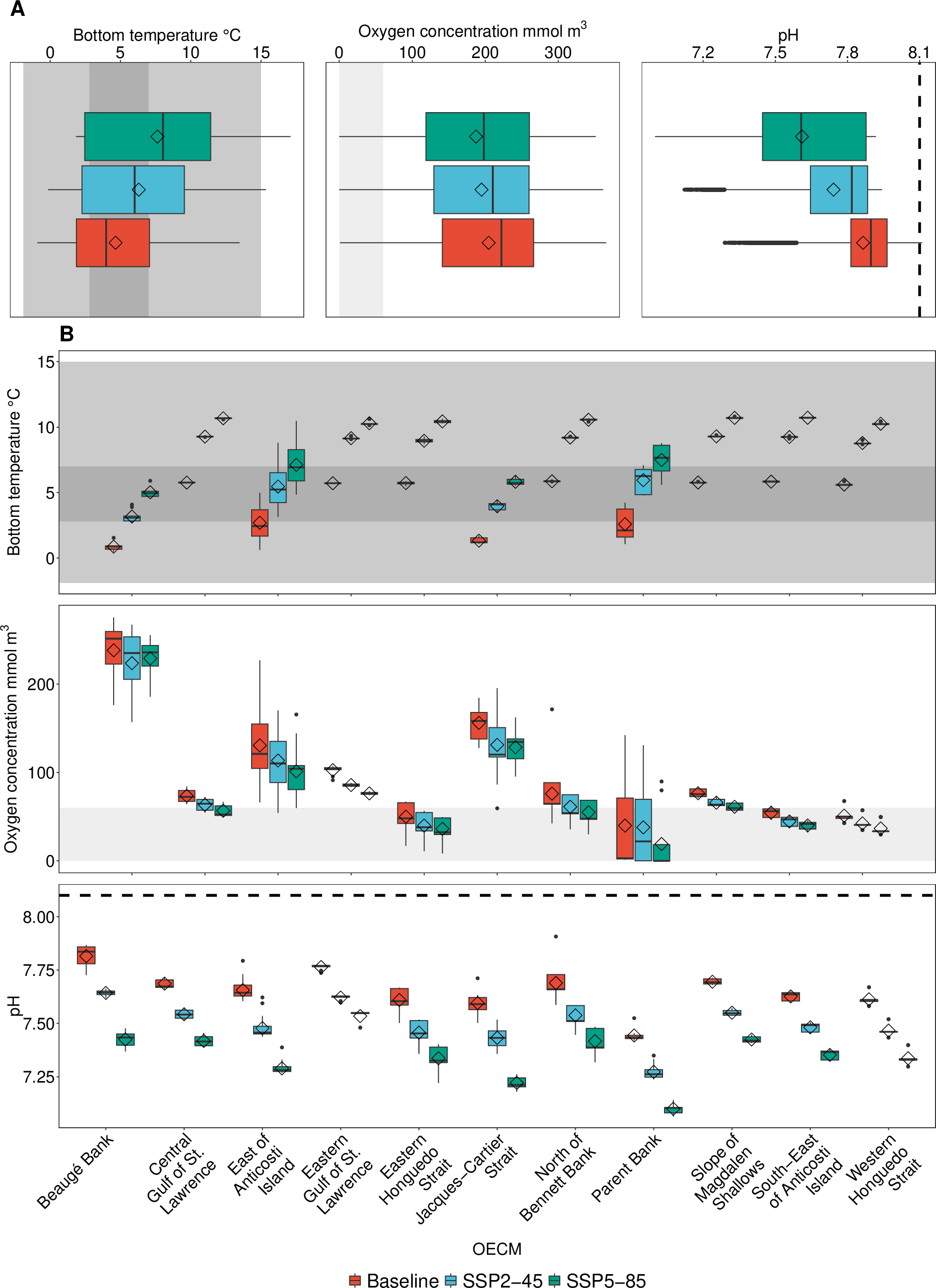

Supplement: S3 Fig — Baseline values include the years 2010–2019; future projections include the time frame 2090–2099 under two shared socioeconomic pathways (SSPs). Dark grey shading indicates the preferred temperature range for adult non-spawning (grey) and spawning (darkgrey) Atlantic halibut (Hippoglossus hippoglossus), Greenland halibut (Reinhardtius hippoglossoides), and redfish (Sebastes mentella). Light grey shading indicates oxygen concentration below 60 mmol m3, the threshold for a marine ecosystem to be considered hypoxic [20]. Dashed black line indicates today’s average ocean pH [59]. Boxplots: upper and lower hinges correspond to the first and third quartiles; upper/lower whiskers extend to the highest/lowest value within 1.5 times the interquartile range; horizontal lines within boxes correspond to the median; diamonds represent the mean; outlier dots represent data beyond the end of the whiskers. (TIF) [file pone.0316754.s003.tif]

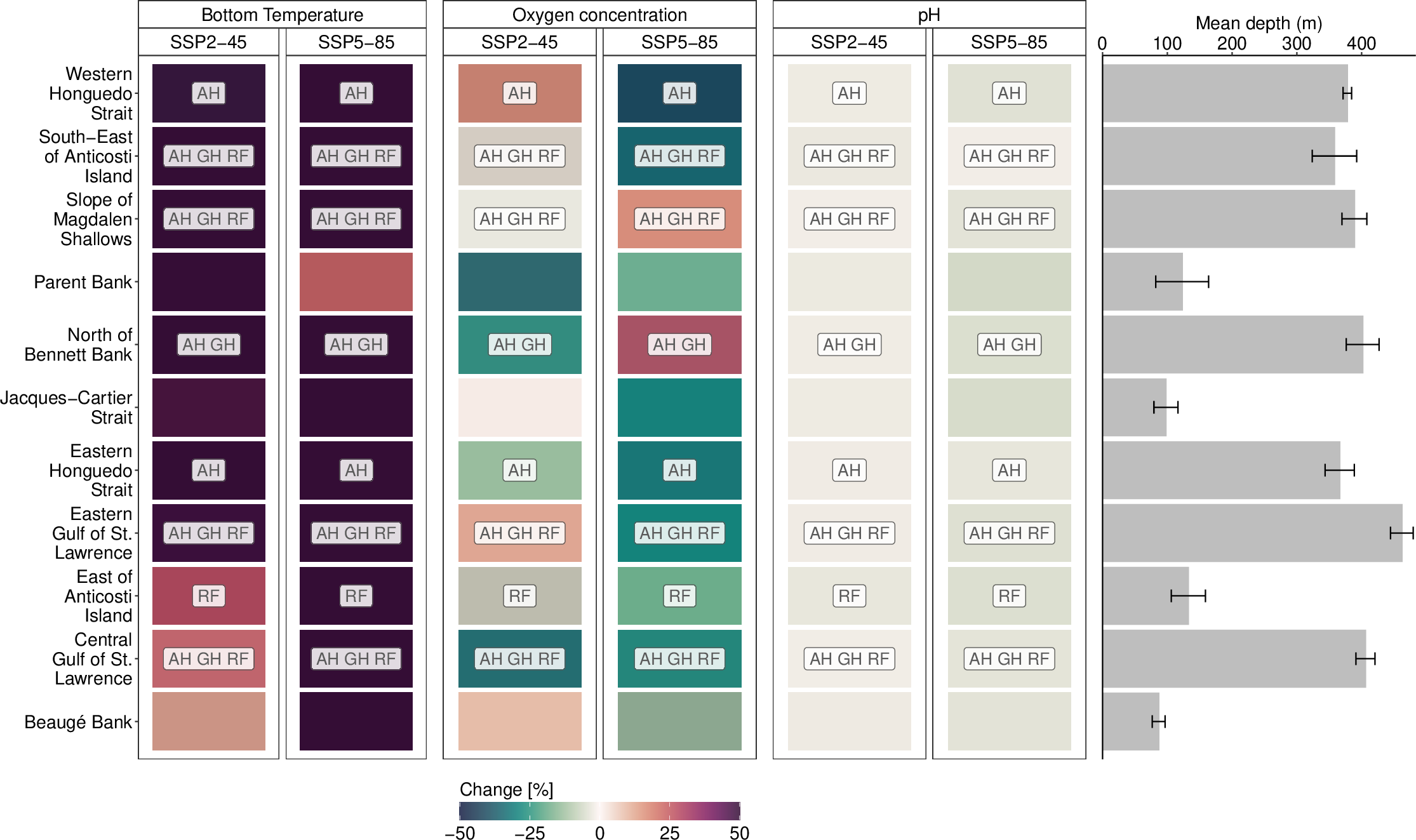

Supplement: S4 Fig — Changes represent values in 2090–2099 relative to the baseline time frame 2010–2019. Labels indicate whether an OECM is overlapping with potential spawning habitat of AH = Atlantic halibut, GH = Greenland halibut, and RF = redfish. Error bars indicate one standard deviation of the mean. (TIF) [file pone.0316754.s004.tif]

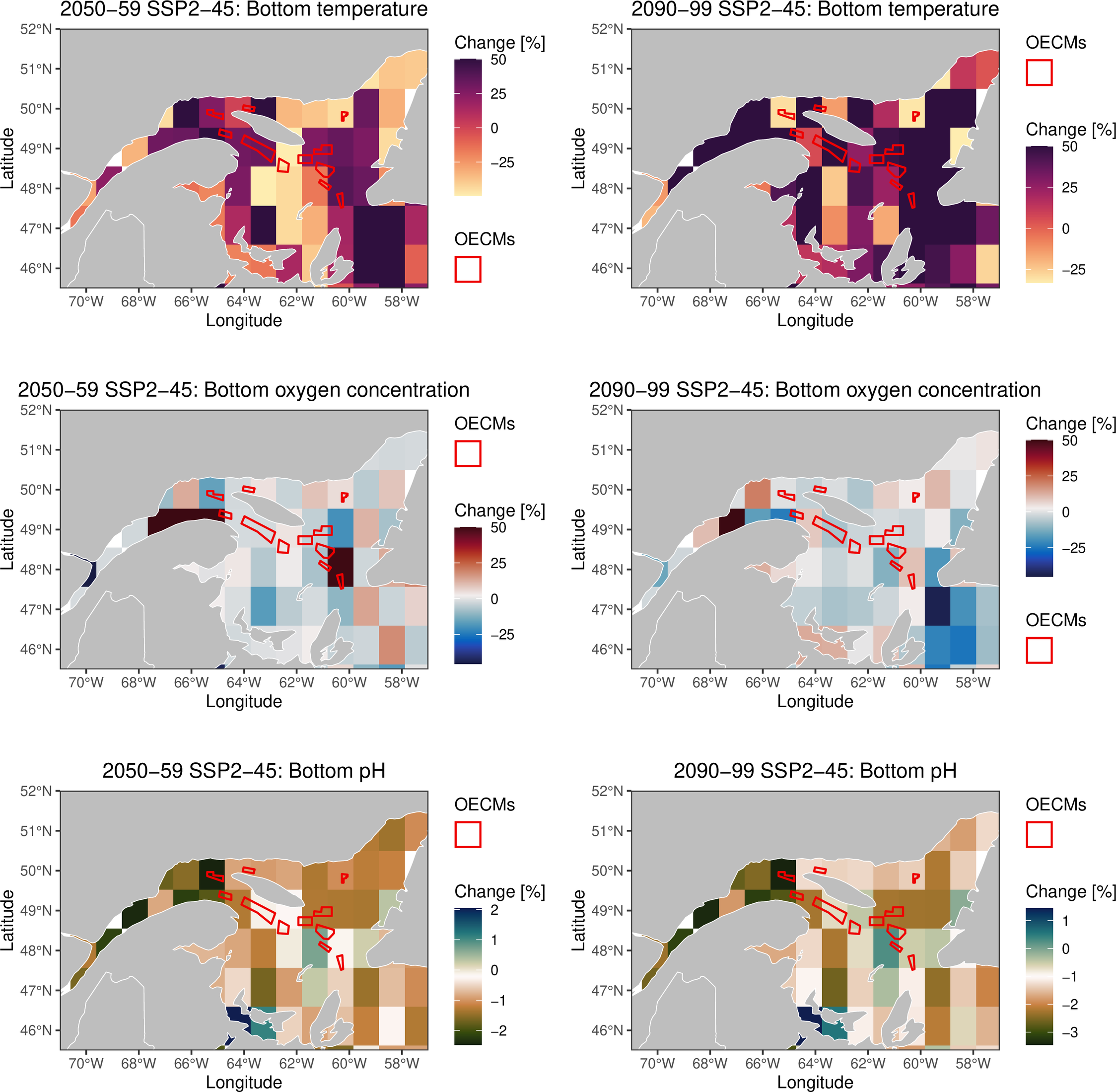

Supplement: S5 Fig — All changes are relative to the baseline condition in 2010–2019. Red outlines denote established Other Effective Conservation Measures (OECMs), retrieved from [45]. Data from Bio-ORACLE v.3.0 [58]. Land shapefiles retrieved from http://www.naturalearthdata.com/. (TIF) [file pone.0316754.s005.tif]

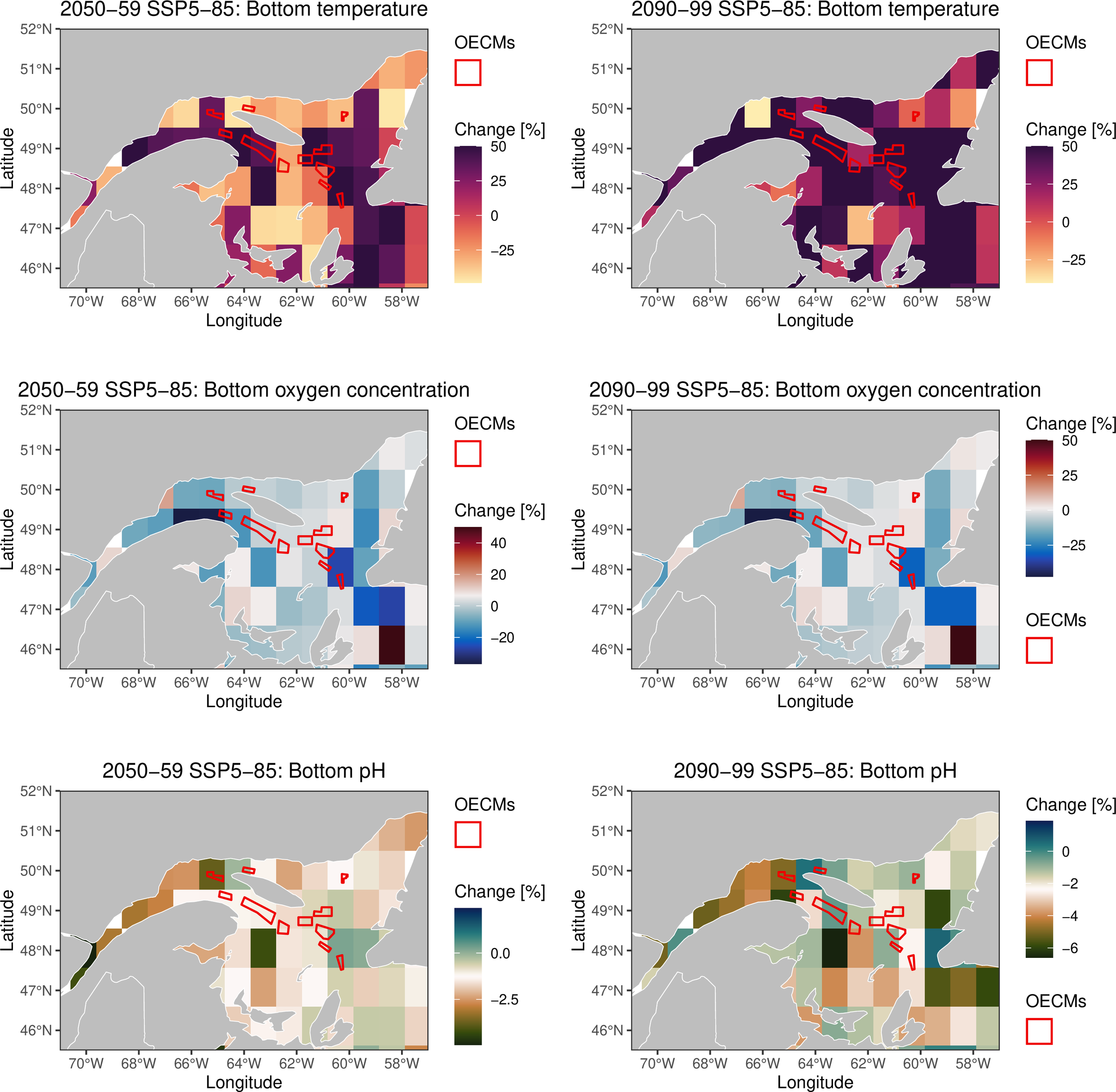

Supplement: S6 Fig — All changes are relative to the baseline condition in 2010–2019. Red outlines denote established Other Effective Conservation Measures (OECMs), retrieved from [45]. Data from Bio-ORACLE v.3.0 [58]. Land shapefiles retrieved from http://www.naturalearthdata.com/. (TIF) [file pone.0316754.s006.tif]
